# Implementing a Virtual Emergency Department: Qualitative Study Using the Normalization Process Theory

**DOI:** 10.2196/39430

**Published:** 2022-09-12

**Authors:** Jennifer Shuldiner, Diya Srinivasan, Justin N Hall, Carl R May, Laura Desveaux

**Affiliations:** 1 Institute for Health System Solutions and Virtual Care Women's College Hospital Toronto, ON Canada; 2 Department of Emergency Services Sunnybrook Health Sciences Centre Toronto, ON Canada; 3 Division of Emergency Medicine Temerty Faculty of Medicine University of Toronto Toronto, ON Canada; 4 Faculty of Public Health and Policy London School of Hygiene and Tropical Medicine London United Kingdom; 5 North Thames Applied Research Collaboration London United Kingdom

**Keywords:** virtual care, emergency department, Normalization Process Theory

## Abstract

**Background:**

COVID-19 necessitated the rapid implementation and uptake of virtual health care; however, virtual care’s potential role remains unclear in the urgent care setting. In December 2020, the first virtual emergency department (ED) in the Greater Toronto Area was piloted at Sunnybrook Health Sciences Centre by connecting patients to emergency physicians through an online portal.

**Objective:**

This study aims to understand whether and how ED physicians were able to integrate a virtual ED alongside in-person operations.

**Methods:**

We conducted semistructured interviews with ED physicians guided by the Normalization Process Theory (NPT). The NPT provides a framework to understand how individuals and teams navigate the process of embedding new models of care as part of normal practice. All physicians who had worked within the virtual ED model were invited to participate. Data were analyzed using a combination of inductive and deductive techniques informed by the NPT.

**Results:**

A total of 14 physicians were interviewed. Participant experiences were categorized into 1 of 2 groups: 1 group moved to normalize the virtual ED in practice, while the other described barriers to routine adoption. These groups differed in their perception of the patient benefits as well as the perceived role in the virtual ED. The group that normalized the virtual ED model saw value for patients (coherence) and was motivated by patient satisfaction witnessed (reflexive monitoring) at the end of the virtual appointment. By contrast, the other group did not find virtual ED work reflective of the perceived role of urgent care (cognitive participation) and felt their skills as ED physicians were underutilized. The limited ability to examine patients and a sense that patient issues were not fully resolved at the end of the virtual appointment caused frustration among the second group.

**Conclusions:**

As further digital integration within the health care system occurs, it will be essential to support the evolution of staff skill sets to ensure physicians are satisfied with the care they are providing to their patients, while also ensuring the technology and process are efficient.

## Introduction

Canadian emergency departments (EDs) endure overcapacity and significant resource constraints [1]. Visits to EDs in Ontario, Canada’s most populous province, have grown by 24.8% in the last decade, rising from 5.1 million in 2009 to 6.5 million in 2019 [2,3]. As ED visits increase and wait times to physician assessment lengthen, resources are increasingly stressed while patient satisfaction decreases [4]. As a result, there is growing attention toward finding alternative patient care options to improve system sustainability [5-7].

Virtual care utilization has increased across health care yet remains underutilized in the ED setting. Virtual care had limited uptake in EDs prior to COVID-19 due to various factors including limited financial compensation, licensure restrictions, and lack of connectivity to resources required to build the system [8]. The COVID-19 pandemic markedly altered patterns of health care utilization in Canada with the introduction and expansion of virtual care. Canadians are accessing physicians through digital technology, and virtual care increased from 1.6% in 2019 to 70.6% in 2020 [9]. Surveys have found that virtual care saves time, improves access, and can be easy to use [10]. In a Canadian survey in May 2020, those who connected with their doctor virtually during COVID-19 reported a 91% satisfaction rate with 46% indicating a preference toward a virtual visit as the first point of contact with their doctor [11].

COVID-19 prompted many emergency care facilities to operationalize virtual care services [12]. The number of Ontario ED visits decreased by 25% in March 2020, indicating that people who should be seeking ED care were not [13]. In response to these trends, Sunnybrook Health Sciences Centre, an academic tertiary/quaternary care hospital in Toronto, Ontario, piloted a virtual ED in December 2020 that connected patients directly to ED physicians. As the first virtual ED to launch in the Toronto area, the design, planning, and implementation were informed by patients and providers of virtual care services in other specialties and emergency services in other regions. To support iterative developments and sustainability, an embedded evaluation was included alongside the pilot launch to address our understanding of whether and how the virtual ED—a complex health care intervention—is actually adopted and sustained as routine practice. The Normalization Process Theory (NPT) [6,14] is a widely used theory of implementation for achieving this understanding and is often used to understand the implementation of eHealth applications [15]. The aim of this study was to use the NPT to understand ED physician experiences of virtual ED implementation and whether and how they were able to integrate a virtual ED alongside in-person operations.

## Methods

### Design

We conducted an NPT-informed qualitative study to explore the implementation of a virtual ED (ED services delivered virtually).

### Normalization Process Theory

Normalization is defined as the embedding of a technology as a routine and taken-for-granted element of clinical practice and focuses on the “work” of implementation. The NPT defines implementation as the “translation of strategic intentions into everyday practices” through collective action and collaborative work. The NPT identifies, characterizes, and explains the mechanisms that motivate and shape implementation processes. In this analysis, we considered the role of 4 implementation mechanisms—coherence (what is the work), cognitive participation (who does the work), collective action (how does the work get done), and reflexive monitoring (how is the work understood) [6,16].

### Context and Setting

Sunnybrook Health Sciences Centre is an adult academic tertiary/quaternary care hospital in Toronto, Ontario, fully affiliated with the University of Toronto. It is a regional trauma, cancer, high-risk maternal, neonatal, neurosurgical, interventional cardiology, and stroke center. The hospital sees 1.3 million patient visits annually with approximately 60,000 ED visits annually. ED visits are funded through an alternate funding agreement with the Ontario Ministry of Health and Long-Term Care.

### The Intervention

In December 2020, Sunnybrook Health Sciences Centre launched a 6-month virtual ED pilot which was continued. From December 2020 to December 2021 the virtual ED had a total of 1987 virtual visits, with a median of 150 visits per month (SD 25). The virtual ED is staffed by ED physicians weekday afternoon and evenings in parallel to the in-person ED. Physicians that worked in the virtual ED voluntarily signed up for the program and completed these shifts in addition to their regular in-person clinical requirements (these shifts were not replacements for their previously agreed to clinical load). Patients self-triaged using a web form via the Sunnybrook Health Sciences Centre website. Patients are advised of potentially appropriate and inappropriate conditions for a virtual visit along with advice of when to consult their family physician as their primary contact for lower acuity concerns. Patients registered online for a same-day appointment at the virtual ED.

A dedicated patient administrative assistant confirmed demographic information and valid health card and created an appointment that was emailed or texted to the patients (based on their stated preference). The assistant also emailed a calendar invitation to the ED physician’s secure hospital email to alert him/her of a new appointment. The patient met with the ED physician via Zoom video and discussed the concern and a plan for moving forward. The administrative assistant helped patients navigate technological difficulties, communicated written instructions and any follow-up investigations or referrals directly with patients, and coordinated patient experience surveys. The ED physicians and administrative assistants used phone, SMS text messaging, and secure email to support their workflow.

Four possible care pathways can result from a virtual ED visit: (1) the patient’s care can be managed during the virtual appointment including potential prescriptions faxed directly to their preferred pharmacy, (2) the patient may be reassured that their issue can be managed through their family physician and does not require urgent care, (3) the patient is scheduled for follow-up for diagnostic imaging such as x-rays or ultrasounds or blood work at the outpatient area of the hospital for the same or next day, or (4) the patient may need to come to the ED for urgent in-person assessment and further investigations.

### Data Collection

Physicians who participated in the virtual ED received an invitation via email from JNH, implementation lead of the virtual ED. Interested physicians contacted the study coordinator who explained the study, provided a study information sheet, and obtained consent. All interviews were conducted by DS and were recorded and transcribed verbatim. We collected descriptive information such as demographic details (ie, gender and age) and data on their ED experience (ie, number of shifts worked per month). The interview guide was structured around the 4 constructs of the NPT to enable exploration of physicians’ experiences of implementing the virtual ED. It was also revised to include issues that emerged as important in early interviews (Multimedia Appendix 1). For example, we began to ask questions around the ED physician’s sense of role and identity as that impacted their sense of legitimation and buy-in into the intervention. Interviews were recorded and transcribed. All physicians received a CAD $50 (US $38.5) e-gift card in remuneration for dedicating their time to an interview.

### Data Analysis

#### Overview

Thematic analysis using inductive and deductive coding was used to describe the manifest and latent content [17]. This approach complemented the research questions by allowing the NPT domains to be integral to the process of deductive thematic analysis while allowing for themes to be derived directly from the data using inductive coding.

#### Directed Content Analysis

A codebook was prepared a priori (Multimedia Appendix 2) and involved adapting the NPT framework to the context of virtual ED and was agreed upon by the study team. All transcripts were coded independently line-by-line by 2 research team members (JS and DS). Deductive codes were compared for the first 3 interviews to achieve consensus and the remaining interviews were coded independently. The first level of coding was deductive based on the NPT domains.

#### Thematic Analysis

Inductive coding was considered on a case-by-case basis. Subsequent levels of coding involved re-examining the content of the codes and narrowing in on more specific elements discovered in the data during coding. Initial themes were then reviewed and refined to ensure that the themes represented the data set as a whole and that no themes were missed or overrepresented.

#### Integrative Analysis

NVivo 12 (QSR International) was used to manage the data set. An initial thematic framework was drafted and was discussed in data analysis workshops among all the authors. The framework underwent several iterations as new issues emerged in the meetings. The final synthesis and interpretation involved considering each theme and subtheme in the context of the whole set of interviews.

The trustworthiness, or credibility, of the study was enhanced by having 2 researchers (JS and DS) working closely together on data analysis. A detailed codebook was produced to ensure uniformity of coding. Meetings with the larger research team throughout the analysis process provided additional insights from experts in emergency medicine, qualitative research, implementation science, and the NPT. This process provided feedback, allowed any shortcomings in the analysis to emerge, and verified the data analysis and interpretation processes [18].

### Ethics Approval

Ethical approval was received from The Research Ethics Board of Sunnybrook Health Sciences Centre (2021-0040-E). All participants consented to participate.

## Results

### Physician Demographics

**Figure 1:**
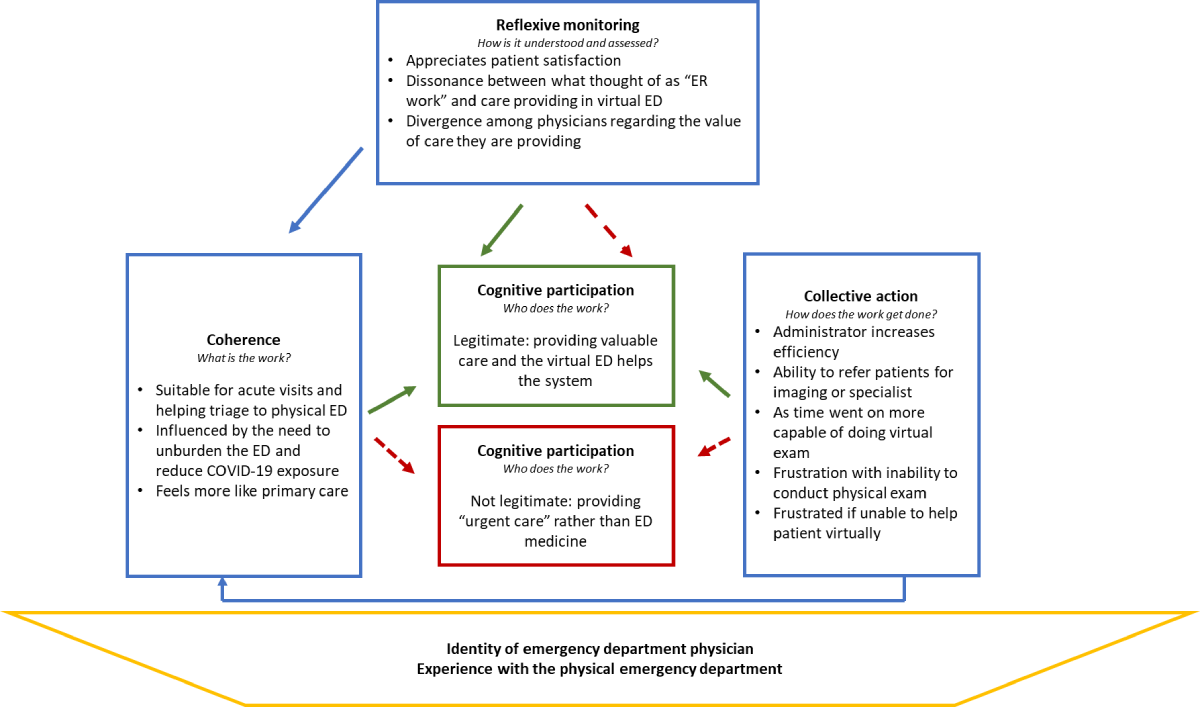
Thematic framework of the normalization process of the virtual ED. ED: emergency department.

We reached out to all physicians who had competed a virtual ED shift (21 physicians) and 14 agreed to be interviewed. Of these, 7 (50%) were female with an average of 16 years of experience (range 6-41 years; Table 1). Our analysis describes 2 pathways for physicians in our study, with 1 group moving to normalize the virtual ED in practice (10/14, 71%), while the other elected to not fully adopt it (4/14, 29%). The first group saw value for patients (coherence) and was motivated by patient satisfaction and the relief witnessed (reflexive monitoring) at the end of the virtual appointment. By contrast, the other group did not find virtual ED work reflective of urgent care (cognitive participation) and felt their skills as ED physicians were underutilized (a denormalization of their role). For them the virtual ED more closely resembled the role of primary care (Figure 1).

**Table 1:** Characteristics of emergency department physicians (n=14).

Characteristics		Value
Age (years), mean (range)		46 (31-67)
**Gender, n (%)**		
	Male	7 (50)
	Female	7 (50)
Experience (years), mean (range)		16 (3-41)
**Number of emergency department shifts per month, n (%)**		
	1-8	4 (29)
	8-15	7 (50)
	15+	3 (21)
**Prior experience with virtual care, n (%)**		
	Yes	6 (43)
	No	8 (57)

### Coherence: Providing Valuable Care to Patients

*Coherence* involves the sense-making work that individuals undertake to bring meaning to a practice. It was reflected in how ED physicians understood the value, responsibilities, tasks, and objectives of the virtual ED. A central value reported by physicians was the support the virtual ED provided for patients on deciding whether an in-person ED visit was necessary. This refers to the work of *differentiation*, where participants sorted and classified the elements of the virtual ED in comparison to the standard in-person model to make sense of whether and how it fits within their understanding of ED care:

I’ve sent a fair number of people to the [physical] emergency department to get evaluated. And what I hear from them mostly is reassurance because they don’t want to go to the emergency department unless they need to. And so, when I talk to them and say, look, here are my concerns...that has been something that the patients have expressed appreciation for, because they don’t want to wait eight hours in the emergency room if they don’t have to.

Physician 11

Physicians described which patient scenarios they felt were most fitting for a virtual ED model, including follow-up from a diagnostic test, mental health issue, or supporting episodic care. While all physicians believed the virtual ED was an efficient and patient-centered model, those who normalized the practice saw value in providing this care themselves while others felt like this was not the best use of their skill set. Physicians described the downstream benefit to the system of easing the burden of in-person care within an already stressed environment, which reduced the psychological pressures on ED physicians.

So the mental health of the people working at the Emergency is going to be better if there’s less people there, because you can’t really focus on the emergencies, rather than thinking, “Oh my goodness, there’s no way I’m going to get through my night shift because there’s 40 people in the waiting room.” Psychologically – that’s a very, very tough hill to climb...that’s easing my mental suffering about the stress of being on that shift.

Physician 3

Some also described how they appreciated being able to see a patient in a more intimate environment without the commotion of the physical ED and the additional context this provided during a care interaction.

While many physicians had concerns around the quality of care within a virtual ED model in its early stages, these were alleviated when the value for patients and physicians became clear. Specifically, patients who were reluctant to seek medical care during the pandemic due to concerns about the risk of in-person exposure now had a mechanism to safely connect with the health system.

I feel stronger about it, now that I see that people are calling in. They have trust in the care that we’re giving. Some people are calling just to get reassurance that they’re OK to stay at home. Some people are calling to get treatment over the virtual emergency care platform...we’re providing successful care and people are pleased with the care they’re receiving. So, we should be doing it.

Physician 2

The group of physicians who elected not to normalize the virtual ED model described how the virtual shifts felt more like delivering primary care. They believed in the value of the care being provided but felt that they were no longer providing ED medicine. Some also speculated that patients were using the virtual ED as a substitute for their family physician to gain quicker access to specialists.

I think what’s happening is that the emergency physician is now turning into a family doctor. I think you have to be careful. You’re giving patients the impression that you are doing family doctor services. I think some people do it because they can then get an appointment with a specialist in the hospital.

Physician 1

### Cognitive Participation: Legitimate Emergency Work for Some But Not for Others

*Cognitive participation* refers to the relational work that people do to engage and commit to a new intervention. We explored how physicians work with others, drive implementation, and see the virtual ED as part of their role. We found that for some engaging in the work of the virtual ED and witnessing the value firsthand was a key element to overcoming initial skepticism and legitimating their role. This *legitimation* involves the work of reflecting on and deciding whether the virtual ED is the right thing to do and a meaningful use of their time and should therefore become part of their routine work.

Initially I was quite sceptical as to how it would work, I’ve never really done virtual care before, and I couldn’t envision patients that I would be able to treat virtually. I thought that any patient would wind up being sent into the emergency department for an evaluation...But now having done it, I actually like it a lot and it does seem useful for patients.

Physician 15

Physicians described that they were able to help patients by advising on the need for an in-person visit, booking a diagnostic test, or a referral to a specialist. This was a shift from their experience of providing value in the physical ED, where they were able to physically examine patients and provide complex care. These care pathways and decision points increased the legitimacy of the virtual ED model among those who normalized it.

However, those physicians who elected not to normalize the virtual ED had concerns over the legitimacy of their role, often feeling that this work was not aligned with the perceived role of an ED physician—a perception that was informed by their training and their prior experiences in providing in-person ED care. These physicians described their belief that any health issue that did not require in-person care was an issue meant for a primary care physician. The virtual model limited their ability to utilize the resources of the ED which led some to feel unfulfilled.

I don’t think that what we’re doing is specific to emergency medicine. Like the job that we signed up for, for emerge is more the acute things that day-to-day stuff with resources. So even being called – like one time I was called on an airplane, is there a doctor on the plane for this patient? And without resources it was quite difficult to take care of the patient because of the nature of our jobs...I don’t feel that the virtual emerge is fulfilling to me in terms of being an emergency physician.

Physician 8

A key distinction was the function of triaging patients versus providing emergency treatment. Where patients required treatment that could be achieved virtually, physicians who elected not to normalize the virtual ED believed these interactions to be within the scope of primary care.

Yeah, it's a lot more like primary care, than emergency care, in what we're providing. It's sort of replacing – emergency triage. We decide if the patient, needs an emergent assessment, but we're not providing any emergency care, but I guess we're using our emergency expertise. Does this person need emergent assessment? But if we're truly treating the patient virtually, it's something that I think is in the realm of primary care, which we also do in the ER, I guess, sometimes. When patients come in with more primary care complaints, we still manage them.

Physician 9

### Collective Action: Physicians Adapted to the Limitations of a Virtual ED Environment

*Collective action* explores how the work of implementing new interventions into practice is done. Both groups needed to navigate a new virtual work environment and consider how to adapt their skill sets and resources to a virtual model. They were able to adapt and learn how to work within the limitations of a virtual care appointment (eg, asking a patient to help with range of motion or a caregiver to support a strength test). Physicians described the efficiency of being able to prepare for the visit by viewing the chief complaint (and sometimes the patient history) prior to initiating the virtual consult. The administrative assistant played an essential function in helping with technological issues, or sending prescriptions or referrals:

The support by the administrative staff that are on, and helping, has been excellent. I have had a couple of different people fulfil that role. And I found in both cases they were very helpful at keeping things running and answering questions.

Physician 3

Physicians also described that their access to the same diagnostic testing or referrals that they were accustomed to in the physical ED was crucial to providing care. This enabled them to practice and provide value as if they were in the physical ED.

So, [in the beginning] the piece that I felt was missing was the comprehensive bloodwork and radiology. So, diagnostic imaging that we hadn’t been offering previously but we’ve started doing that. And so, I feel that really allows them to “come” to the Virtual Emergency Department and we can say to them, “Yeah. We’re going to order this diagnostic imaging today. Yes, we’re offering bloodwork.

Physician 2

### Reflexive Monitoring: Reflecting on Value From Patient Interactions

*Reflexive monitoring* refers to how individuals work together to appraise the intervention and how it is working. Physicians described evaluating success and impact through feedback from their patient interactions. For those who normalized the virtual ED, the feedback they received from patients was formative of how they viewed their role in the virtual ED. The immediate feedback provided by patients gave them the assurance that they were providing valuable care and reinforced the legitimation of their role. For example, some physicians were surprised by the great value of providing patients guidance on whether in-person ED visit was warranted.

Do I need to go to the Emerge for this? Can this wait? Should I make an appointment?” And that advice – I don’t think we realize how valuable it is.

Physician 4

This immediate positive feedback reinforced the coherence of the virtual ED physicians and their views of how this work aligned with their role as an emergency physician. They also saw value in providing patients with access to emergency services without having to send them into the physical ED and connecting them directly with outpatient follow-up. Although they recognized that the care they were providing was different than what they were used to, the physicians who normalized the virtual ED were reinforced by the positive impact they were having on patients.

All the patients I’ve seen, they’ve pretty much all given me positive feedback saying what a wonderful service this is and I think we’ve also added things to the virtual service that has made it be more applicable to emerge, like being able to order an X-Ray for a patient virtually so they can go and get an X-Ray done or get an ultrasound done the next day rather than just sending them into the emerge for a visit to do that is really useful. And being able to have access to our regular outpatient follow up options.

Physician 14

The physicians who elected not to normalize the virtual ED still recognized its positive impact; however, this value was not strong enough to overcome the perception that the virtual ED was not a legitimate use of their emergency skills.

I’m not offering a lot of extra value, seeing them in the [virtual] Emergency Department. So, there is a bit of skepticism around the sustainability of whether emergency physicians will continue to have a role here or whether we should think of expanding primary care for these types of problems.

Physician 3

## Discussion

### Principal Findings

Our work explored the dynamic implementation process of a virtual ED to identify the elements that contributed to the virtual model becoming normalized for some physicians but not others. We found that cognitive participation and legitimation played a key role in normalizing the virtual model. For some, the satisfaction of providing quality and beneficial care for patients overshadowed any concerns of not using their skills as an emergency physician to their fullest potential. For others, the limited ability to examine patients and a sense that patient issues were not fully resolved at the end of the virtual appointment caused frustration. These physicians signed up for fewer shifts and did not experience the continuously evolving model and capabilities of the virtual ED platform. The virtual ED was normalized as an organizational operating model, as it was routinely incorporated into practice. However, some participants saw that their professional roles and skills as being denormalized. This resulted in *relational restructuring*—where there was a discordance between value for patients and their professional identity—which led them to opt out of the virtual ED model as participation was voluntary. Other physicians adapted to the *normative restructuring* by shifting their perspective on whether and how their unique skills set added value amid shifting standards and workflows within the virtual ED as compared with the in-person model (eg, saw value in helping with triage to the in-person ED).

For those physicians who elected not to normalize the virtual ED, relational pathways between legitimation and coherence were not present. These physicians felt the care they were providing did not fit with their identity as an emergency physician, highlighting the influence of social norms in the successful uptake of a virtual ED model. Social norms theory posits that individuals are characterized by a variety of context-dependent connections, social roles, and rules in the form of norms and conventions [19,20]. Therefore, to promote virtual emergency medicine, we found that it was important to consider the importance of the culture and norms of physicians’ professional identity and ensure that in-person care translates to the virtual shift. Similarly, research has shown that predictors of physicians’ intention to use telemedicine in their clinical practice are influenced by their perception of what the social groups to which they belong expect from them [21-23].

It is not surprising that the pivot to a virtual ED model is very dramatic for a specialty that is trained and habituated to working in a fast-paced and high-stakes environment. The virtual ED required ED physicians to restructure their behavior and how they practiced medicine. Their professional identities as ED physicians felt incongruent with the care they were providing in the virtual ED. This had a great impact on legitimation, and the value created for patients was not sufficient to overcome the perceived shift in professional identify for a subset of physicians. The role of professional identity in the normalization of complex interventions may be rooted in a perceived threat of professional traditions, which has been a driver of physician resistance to virtual care more broadly [24-26]. Similarly, ensuring staff are supported in developing the necessary skill sets for virtual models is an important element of supporting normalization [27].

The COVID-19 pandemic has transformed the way health care is delivered, with various sectors providing more care through technology. Emerging evidence has documented physician views about the challenges of rapidly implementing telemedicine [28-31]. A survey among nephrologists reported increased access for patients, but concerns with proper physical examination, monitoring, and education of patients. They also reported less job satisfaction and sense of connection with patients [32]. Many studies focused on family physicians and generally, primary care clinicians have found virtual care acceptable, improves access and quality of care [33-36], and provides them with flexibility [36]. Studies have also reported that physicians felt it was a useful addition, saves time, and can enhance patient care. Further, some even preferred to provide follow-up for their patients by telemedicine rather than face-to-face clinics [35]. However, they have also noted changes to physician-patient interactions [34]. This physician population was generally positive and did not have the same concerns regarding a shift in professional identity or the type of medicine they were practicing.

### Limitations

This evaluation took place as iterative improvements to the virtual ED were taking place and therefore experiences of the virtual ED were different for some physicians (eg, unavailability of ordering diagnostic imaging). Participation in the virtual ED was voluntary and physicians were interviewed at a single point in time, limiting our ability to explore whether experience over time shifted engagement with or perceptions of the virtual ED model. Also, we interviewed 67% (14/21) of emergency physicians and we do not know how the experiences of those we did not interview would impact our findings. The study included physicians from a single ED and it is unclear whether or how these results would generalize to other settings. Finally, while the COVID-19 pandemic was the catalyst for the virtual ED model, it has created artificial circumstances under which the model initially operated. Future work should explore whether and how the model and its use evolve under normal operating conditions.

### Conclusions

The rapid implementation of an innovative model for urgent care delivery provided an opportunity to understand how ED physicians integrate virtual care and the factors that influence uptake. Understanding the implementation of complex interventions is an important challenge for health care administrators and policy makers who must make decisions regarding the intervention and eventually scale and spread. The NPT is useful for exploring a greater understanding from participants as to how they make sense of and internalize a new technology, which in turn may help address and mitigate resistance from health professionals. Specifically, it highlighted the need to communicate how a new intervention aligns with professional identity and to communicate whether and how the creation of value is different from current experiences. As further digital integration within the health care system occurs, it will be essential to support the evolution of staff skill sets to ensure physicians are satisfied with the care they are providing to their patients, while also ensuring the technology and process are efficient.

## Appendix

Multimedia Appendix 1 Interview guide.

Multimedia Appendix 2 Normalization process theory coding framework.

## Abbreviations

ED: emergency department
NPT: Normalization Process Theory

## Availability of Data and Materials

Data collected in this study are interview transcripts and excerpts and are available upon request, as per Research Ethics Board ethics.

## References

[1] Affleck A, Parks P, Drummond A, Rowe BH, Ovens HJ (2013). Emergency department overcrowding and access block. CJEM.

[2] Canadian Institute for Health Information (CIHI) (2019). NACRS Emergency Department Visits and Length of Stay, 2018–2019. CIHI.

[3] Health Quality Ontario (2016). Under Pressure: Emergency department performance in Ontario. Health Quality Ontario.

[4] Sonis JD, Aaronson EL, Lee RY, Philpotts LL, White BA (2018). Emergency Department Patient Experience: A Systematic Review of the Literature. J Patient Exp.

[5] Canadian Medical Association (2011). Report of the Advisory Panel on Resourcing Options for Sustainable Health Care in Canada to the Canadian Medical Association.

[6] May C, Finch T (2009). Implementing, Embedding, and Integrating Practices: An Outline of Normalization Process Theory. Sociology.

[7] (2019). Hallway Health Care: A System Under Strain -- 1st Interim Report from the Premier’s Council on Improving Healthcare and Ending Hallway Medicine. Government of Ontario.

[8] Canadian Medical Association (CMA) (2019). Virtual care in Canada: Discussion paper. CMA.

[9] Bhatia RS, Chu C, Pang A, Tadrous M, Stamenova V, Cram P (2021). Virtual care use before and during the COVID-19 pandemic: a repeated cross-sectional study. CMAJ Open.

[10] Canada Health Infoway (2016). Virtual Visits in British Columbia: 2015 Patient Survey and Physician Interview Study. Canada Health Infoway.

[11] Canadian Medical Association (CMA) (2020). What Canadians Think About Virtual Care. CMA.

[12] Rosenfield D, Lim R, Tse S (2021). Implementing virtual care in the emergency department: building on the pediatric experience during COVID-19. CJEM.

[13] Canadian Institute of Health Information (CIHI) (2020). NACRS emergency department visits and lengths of stay. CIHI.

[14] Campbell SM, Hann M, Hacker J, Burns C, Oliver D, Thapar A, Mead N, Safran DG, Roland MO (2001). Identifying predictors of high quality care in English general practice: observational study. BMJ.

[15] Mair FS, May C, O’Donnell C, Finch T, Sullivan F, Murray E (2012). Factors that promote or inhibit the implementation of e-health systems: an explanatory systematic review. Bull World Health Organ.

[16] May C, Rapley T, Finch T, Nilsen P, Birken S (2020). Normalization Process Theory. International Handbook of Implementation Science.

[17] Braun V, Clarke V (2006). Using thematic analysis in psychology. Qualitative Research in Psychology.

[18] Shenton AK (2004). Strategies for ensuring trustworthiness in qualitative research projects. EFI.

[19] Therborn G (2016). Back to Norms! on the Scope and Dynamics of Norms and Normative Action. Current Sociology.

[20] Cialdini R, Kallgren C (1991). A focus theory of normative conduct: A theoretical refinement and reevaluation of the role of norms in human behavior. Advances in Experimental Social Psychology.

[21] Gagnon M, Godin G, Gagné C, Fortin J, Lamothe L, Reinharz D, Cloutier A (2003). An adaptation of the theory of interpersonal behaviour to the study of telemedicine adoption by physicians. International Journal of Medical Informatics.

[22] Kim J, DelliFraine JL, Dansky KH, McCleary KJ (2010). Physicians' acceptance of telemedicine technology: an empirical test of competing theories. IJISCM.

[23] Vallo Hult Helena, Hansson A, Gellerstedt Martin (2020). Digitalization and Physician Learning: Individual Practice, Organizational Context, and Social Norm. J Contin Educ Health Prof.

[24] Mullen-Fortino M, DiMartino J, Entrikin L, Mulliner S, Hanson CW, Kahn JM (2012). Bedside nurses' perceptions of intensive care unit telemedicine. Am J Crit Care.

[25] Ferlie E, Fitzgerald L, Wood M, Hawkins C (2005). The Nonspread of Innovations: the Mediating Role of Professionals. AMJ.

[26] Armfield N, Donovan T, Smith Anthony C (2010). Clinicians' perceptions of telemedicine for remote neonatal consultation. Stud Health Technol Inform.

[27] Murray E, Burns J, May C, Finch T, O'Donnell C, Wallace P, Mair F (2011). Why is it difficult to implement e-health initiatives? A qualitative study. Implement Sci.

[28] Barney A, Buckelew S, Mesheriakova V, Raymond-Flesch M (2020). The COVID-19 Pandemic and Rapid Implementation of Adolescent and Young Adult Telemedicine: Challenges and Opportunities for Innovation. J Adolesc Health.

[29] Srinivasan M, Asch S, Vilendrer S, Thomas SC, Bajra R, Barman L, Edwards LM, Filipowicz H, Giang L, Jee O, Mahoney M, Nelligan I, Phadke AJ, Torres E, Artandi M (2020). Qualitative Assessment of Rapid System Transformation to Primary Care Video Visits at an Academic Medical Center. Annals of Internal Medicine.

[30] Latus-Olaifa Olushola, Norman GJ, Kurliand M, Slaboda JC, Abrashkin KA, Smith KL, Pekmezaris R, Rhodes K (2019). Not Yet Ready for Prime Time: Video Visits in a Home-Based Primary Care Program. J Am Geriatr Soc.

[31] Donelan K, Barreto E, Sossong Sarah, Michael Carie, Estrada Juan J, Cohen Adam B, Wozniak Janet, Schwamm Lee H (2019). Patient and clinician experiences with telehealth for patient follow-up care. Am J Manag Care.

[32] Heyck Lee S, Ramondino S, Gallo K, Moist LM (2022). A Quantitative and Qualitative Study on Patient and Physician Perceptions of Nephrology Telephone Consultation During COVID-19. Can J Kidney Health Dis.

[33] Gold KJ, Laurie AR, Kinney DR, Harmes KM, Serlin DC (2021). Video Visits: Family Physician Experiences With Uptake During the COVID-19 Pandemic. Fam Med.

[34] Gomez T, Anaya YB, Shih KJ, Tarn DM (2021). A Qualitative Study of Primary Care Physicians’ Experiences With Telemedicine During COVID-19. J Am Board Fam Med.

[35] Alakeel R, Alaithan A, Alokeil N, Kofi M (2021). Family physician's perception towards virtual care during COVID-19 pandemic: A cross-sectional study. J Family Med Prim Care.

[36] Breton M, Sullivan EE, Deville-Stoetzel N, McKinstry D, DePuccio M, Sriharan A, Deslauriers V, Dong A, McAlearney AS (2021). Telehealth challenges during COVID-19 as reported by primary healthcare physicians in Quebec and Massachusetts. BMC Fam Pract.

