# Supplementary material for: Implementing a Virtual Emergency Department: Qualitative Study Using the Normalization Process Theory
Source: JMIR Hum Factors. 2022 Sep 12;9(3):e39430. doi: 10.2196/39430 (PMC9513685; doi:10.2196/39430)
Supplement: Multimedia Appendix 1 [file humanfactors_v9i3e39430_app1.docx]

**Multimedia Appendix 1. Interview guide.**

Questions

1. What did you think when you first heard about the virtual ED? [Coherence]
   1. Have your thoughts changed over time?
2. What conversations were had within Sunnybrook and among your colleagues about the virtual ED?
   1. Do you feel training and resources to support the virtual ED are sufficient? [Contextual integration + Skill set workability]
3. Can you tell me about your experiences participating in care delivery via the virtual ED? [Cognitive Participation]
   1. Can you describe any adaptations you have had to make to make the virtual ED work? [Cognitive Participation-enrollment]
4. Can you describe the logistics of using the platform to bring patients into the ED? [Interactional Workability]
5. Was the virtual ED useful for providing care? [Reflexive Monitoring- Individual appraisal]
   - 1. What went well for you and your colleagues?
     2. What do you think was challenging for you and your colleagues?
6. Can you tell me about using the platform to schedule a visit with the family doctor?
   1. What went well for you and your colleagues? [Reflexive Monitoring- Individual appraisal]
   2. What was challenging for you and your colleagues?
7. Can you tell me about a patient visit that went well? A visit that did not go well?
8. Do you find the virtual ED worthwhile? [Coherence-Internalization]
   1. Is it efficient use of your time? [Collective Action- Relational Integration]
   2. Is it helpful for your patients? [interactional workability]
   3. Do you feel your perspective aligns with your colleagues’? [Reflexive Monitoring - Communal appraisal]
   4. For which types of patients or patient complaints do you feel the virtual model is most appropriate?
9. How do you envision a virtual ED model fitting within broader ED services? What would be the key functions/components of this model? [Cognitive Participation- Legitimation]
10. What issues come to mind for you when thinking about the sustainability of a virtual ED in practice? [Reflexive Monitoring- Individual appraisal]
11. How do you think work should be allocated in the virtual ED? What skills are needed and how should they be organized? [Collective Action- Skill set Workability]
12. Overall: how do you think implementation of the virtual emergency department worked for the ED?
13. What are the current barriers to effective care with the virtual emergency department?
14. What, if any, are some changes you would make? [Reflexive Monitoring-Reconfiguration]
