# Supplementary material for: Implementing a Virtual Emergency Department: Qualitative Study Using the Normalization Process Theory
Source: JMIR Hum Factors. 2022 Sep 12;9(3):e39430. doi: 10.2196/39430 (PMC9513685; doi:10.2196/39430)
Supplement: Multimedia Appendix 2 [file humanfactors_v9i3e39430_app2.docx]

**Multimedia Appendix 2. Normalization process theory coding framework.**

|  |  | **Definition** |
| --- | --- | --- |
| **Coherence** | **Differentiation** | When ED physicians/staff use the virtual platform, how do they differentiate between an in person visit and a virtual visit? |
|  | **Communal specification** | How do ED physicians/Staff integrate the virtual ED into the regular ED (shared understanding)? What is their shared understanding of the aims, objectives and practices of the VED? |
|  | **Individual specification** | How do ED physicians/staff understand their specific tasks and responsibilities in the virtual ED? |
|  | **Internalization** | How do ED physicians/staff understand the value, benefits and importance of the virtual ED? |
| **Cognitive Participation** | **Initiation** | How do ED physicians/staff drive the implementation of the virtual ED forward and get others involved? |
|  | **Enrolment** | Do ED physicians/staff work with colleagues in new ways to further/augment their participation in the virtual ED; how? |
|  | **Legitimation** | How do ED physicians/staff believe participating in the virtual ED is a legitimate part of their role and that they contribute to the implementation of the virtual ED? |
|  | **Activation** | Will the ED physicians/staff work continue their support of the virtual ED; how? |
| **Collective Action** | **Interactional Workability** | How do the ED Physicians/staff work with each other and with other elements of their ER work? |
|  | **Relational Integration** | How do they build accountability and maintain confidence in the work they do in the virtual ED?  Do the ED physicians/staff have confidence in others’ ability to use the virtual ED? |
|  | **Skill set Workability** | Do the ED physicians/staff feel/think that work is assigned to those with the appropriate skills to manage [specific aspects] the virtual ED?  Do the ED physicians/staff feel/think that sufficient training has been provided to enable use of the virtual ED? |
|  | **Contextual Integration** | Do the ED physicians/staff feel/think that sufficient resources (i.e., government, hospital) are available to support the virtual ED?  Do the ED physicians/staff feel/think that management adequately supports the virtual ED? |
| **Reflexive Monitoring** | **Systematization** | How do the ED physicians/staff determine if the work they do is successful/useful? What have they found?  Are the ED physicians/staff aware of the impact/results/feedback about the virtual ED? |
|  | **Communal appraisal** | Do the ED physicians/staff feel the virtual ED is worthwhile/valuable? |
|  | **Individual appraisal** | Does the ED physicians/staff value the impact the virtual ED has had on their practice/work? |
|  | **Reconfiguration** | Do ED physicians/staff believe that feedback about the virtual ED can help improve it in the future?  Do ED physicians/staff feel/think they can modify the way they work going forward with/because of the virtual ED?  How do their appraisals lead to changing procedures or modifying practices? |
